# Brain Regions Associated to a Kinesthetic Illusion Evoked by Watching a Video of One's Own Moving Hand

**DOI:** 10.1371/journal.pone.0131970

**Published:** 2015-08-19

**Authors:** Fuminari Kaneko, Caroline Blanchard, Nicolas Lebar, Bruno Nazarian, Anne Kavounoudias, Patricia Romaiguère

**Affiliations:** 1 Laboratoire de Neurosciences Intégratives et Adaptatives, NIA UMR 7260, FR3C FR3512, Aix Marseille Université, CNRS, Marseille, France; 2 Institut des Neurosciences de la Timone, INT UMR 7289, IRMf Center, Aix Marseille Université, CNRS, Marseille, France; Birkbeck, University of London, UNITED KINGDOM

## Abstract

It is well known that kinesthetic illusions can be induced by stimulation of several sensory systems (proprioception, touch, vision…). In this study we investigated the cerebral network underlying a kinesthetic illusion induced by visual stimulation by using functional magnetic resonance imaging (fMRI) in humans. Participants were instructed to keep their hand still while watching the video of their own moving hand (Self Hand) or that of someone else's moving hand (Other Hand). In the Self Hand condition they experienced an illusory sensation that their hand was moving whereas the Other Hand condition did not induce any kinesthetic illusion. The contrast between the Self Hand and Other Hand conditions showed significant activation in the left dorsal and ventral premotor cortices, in the left Superior and Inferior Parietal lobules, at the right Occipito-Temporal junction as well as in bilateral Insula and Putamen. Most strikingly, there was no activation in the primary motor and somatosensory cortices, whilst previous studies have reported significant activation in these regions for vibration-induced kinesthetic illusions. To our knowledge, this is the first study that indicates that humans can experience kinesthetic perception without activation in the primary motor and somatosensory areas. We conclude that under some conditions watching a video of one's own moving hand could lead to activation of a network that is usually involved in processing copies of efference, thus leading to the illusory perception that the real hand is indeed moving.

## Introduction

Kinesthetic sensations usually result from movements, whether voluntarily executed or passively imposed. It is therefore difficult to discriminate which components pertain to the motor execution command and which pertain to sensory feedback. In studies of kinesthesia, it is thus of interest to find situations in which participants experience sensations of movement while they are not actually moving. Such kinesthetic illusions can be elicited by the stimulation of several sensory modalities, including muscle proprioception [1–5], touch [6,7], and vision [8–11].

Several studies employing imaging techniques investigated which cortical areas underlie kinesthetic illusions induced by proprioceptive stimulation. It is now accepted that movement illusions induced by proprioceptive stimulation arise because the stimulation activates the muscle spindles in a similar way to when the muscle actually stretches thus simulating the sensory feedback resulting from a real movement [2,4,12,13]. In a positron emission tomography (PET) study, Naito et al. [14] suggested that activated cortical areas were all motor areas. A few years later, studies using functional magnetic resonance imaging (fMRI) showed that the perception of a kinesthetic illusion elicited by proprioceptive stimulation [7,15–20] was associated with activation in the Motor and Premotor cortices, the Supplementary Motor Area, the Inferior Parietal Lobule, the Cingulate Motor areas, and the Cerebellum. Using magnetoencephalography we confirmed that the beginning of kinesthetic sensations was related to the activation of the Posterior Parietal cortex as well as of the Primary Motor cortex [21].

Illusory sensations of movement have also been reported when a moving tactile stimulation is applied under the palm of the stationary hand of the participants, giving them the feeling that their hand is moving in the opposite direction [7,11]. In an fMRI study, Kavounoudias et al [7] showed that kinesthetic illusions of clockwise hand rotations elicited either by a tactile or a proprioceptive stimulation applied to the right hands of healthy volunteers, were accompanied by the activation of very similar cerebral networks that included the same cortical and subcortical sensorimotor areas as previously reported for vibration induced kinesthetic illusions.

So, there is now widespread evidence that kinesthetic illusions induced by proprioceptive or tactile stimuli rely on a cerebral network of sensory and motor areas similar to that activated during active movement. However, less is known about the neural substrates of visually-induced illusions of movement. The most well-known movement illusion induced by visual stimulation is the vection phenomenon in which a visual flow at continuous velocity elicits the feeling that the whole body is moving in the opposite direction [22,23]. Such vections that relate to displacements of the whole body in space activate mainly visuo-vestibular regions [24–30].

However, visual stimulation can also induce illusions of limb movements [8–11], although the neural mechanisms giving rise to those illusions are still unclear. Over the last ten years, a particular visually-induced illusion, the mirror illusion, has been extensively studied because it is considered as a tool for motor rehabilitation purpose particularly to promote recovery from hemiparesis and hemiplegia [31,32]. In this paradigm, participants place their affected hand behind a mirror. Their other, healthy hand is placed so that it appears on the mirror in alignment with their affected arm. When they move their healthy hand while looking at the mirror, participants get the visual impression that their affected hand is moving. In some instances, particularly when they are instructed to produce bi-manual motor commands, participants can feel that their paralyzed or even absent hand is moving. Although several hypotheses have been proposed, the underlying neural mechanisms are still unknown.

The difficulty lies in the need to disentangle the neural mechanisms of the illusion from those of the bi-manual coupling and those of the actual movement execution by the contralateral hand. Therefore, to identify the cerebral network associated to a visually-induced kinesthetic illusion, we used a paradigm in which kinesthetic illusions can be induced using visual stimulation in the absence of any real movement. In previous experiments, we found that when participants watched videos of a hand the first finger of which was slowly moved on a screen placed over their own hand, they felt the illusory sensation that their own first finger moved [8,9].

In the present study we adapted this paradigm to the constraints of fMRI. We tested 14 participants in a 3T fMRI scanner while watching the video of their own moving hand (Self Hand) or that of someone else's moving hand (Other Hand). In the Self Hand condition, participants experienced an illusory sensation that their hand was moving whereas the Other Hand condition did not induce any kinesthetic illusion. The contrast between the Self Hand and Other Hand conditions has been examined to determine brain regions associated to movement illusions induced by purely visual stimulation.

## Materials and Methods

### Participants

24 healthy right-handed participants (5 males) participated to the present experiment. None of them had a history of neurological or psychiatric illness. The degree of handedness for each participant was determined prior to inclusion on the basis of the Edinburgh Inventory (all above 75; mean 94.6; [33]). Participants were paid for their participation. Among the 24 participants tested, 9 were not included in the fMRI experiment because they did not report any illusion (7) or the latency of their illusion remained higher than 2 seconds after several training sessions (2). The remaining 15 participants (4 males), aged between 20 and 40 (mean 26.33, ± 6.74 SD) were included in the fMRI experiment.

### Ethics statement

The present study was approved by the local ethics committee (Comité de Protection des Personnes Sud Méditerranée 1 #11/14). All participants included in the present study were given written information and signed a consent form before the experiment.

### Experimental set-up

**Fig 1:**
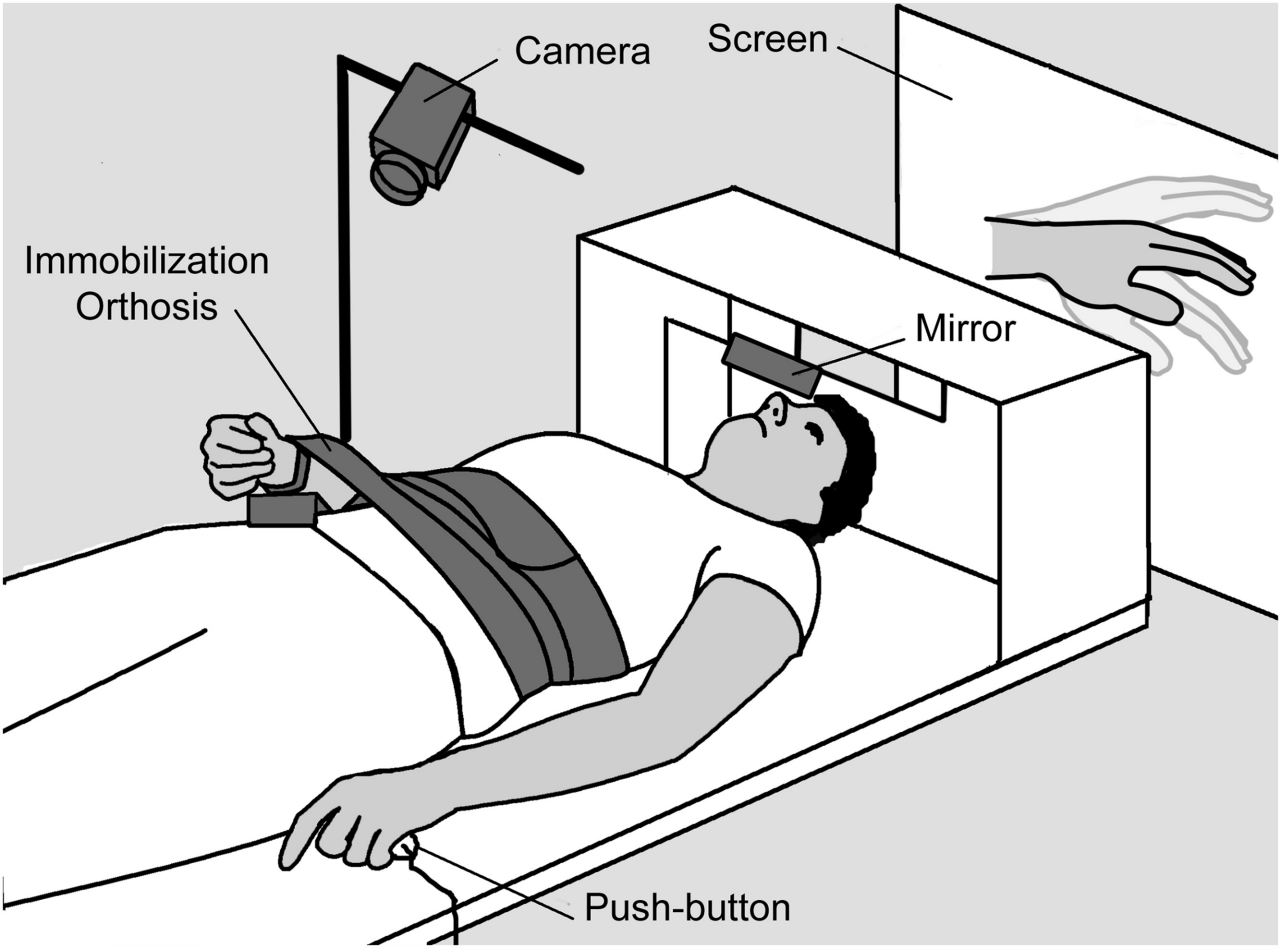
Experimental set-up. The subject's right arm was held by a soft immobilization and he/she could see their right hand through a webcam the output of which was projected onto a screen behind them. A mirror placed before their eyes allowed vision of the screen. They held a custom made push-button with their left hands.

Participants lay in a supine position, their right wrist resting in a semi-prone position on their mid-torso. Their right arm was held by a soft immobilization to ensure the position was constant and the arm relaxed throughout the experiment (Fig 1). Participants could see their right hand through a webcam the output of which was projected onto a screen behind them. The hands were presented as if they were seen from above with an egocentric point of view. A mirror placed before their eyes allowed vision of the screen. At first they simply moved their hand freely for a few minutes to adjust to the point of view. When they reported being sure that they were seeing their own hand on-line, they were asked to perform a series of slow wrist extension-flexion movements that was recorded through the webcam and saved on disk. The pace of the wrist movements was guided by a metronome to calibrate the movements of all the participants at a rate of 0.16 Hz, i.e., one extension-flexion movement every 6 seconds. Participants were then instructed to keep their hand still while watching videos of their own moving hand or of someone else's hand in the same position and moving at the same speed as their own. After a delay, they experienced an illusory kinesthetic sensation that their hand was actually moving when watching the video of their own hand. The latency of the kinesthetic illusion decreased when the experiment was repeated. Before the fMRI experiment, each participant underwent a few training sessions on different days, so that the latency of the illusion was under 2 s. This value was chosen so as to optimize fMRI design. Indeed, even with a block length of 12 s the illusion would still be present for most of the block. During the training session both videos (self and other hands) were presented in equal numbers, and with the same duration. The participant held a custom made push-button to indicate the onset of the kinesthetic illusion for each trial during the training sessions.

### Stimuli

Stimuli were managed using the Labview software package (National Instruments Corporation, Austin, TX, USA). They were delivered to a high luminance LCD projector, back projected onto a frosted screen positioned at the back end of the MRI tunnel, and viewed by the participants through a mirror.

Target stimuli for the two main conditions consisted of 12 s videos of human hands performing slow extension-flexion movements. In the Self Hand condition videos were of the participant's right hand. In the Other Hand condition videos were of someone else's right hand. The Other Hand video was chosen so that the hand was in the same position as the participant's but looked different. Participants were presented with all the videos we had with other people's hands that were in the exact same position (i.e. fingers slightly flexed or fully extended depending on the position that they spontaneously adopted when asked to perform slow extension-flexion movements). Some of these videos did elicit illusions of movement, when the other hand was too similar to that of the participant, so participants were told to pick the hands that they thought were the more different from their own. There were no objective criterion, participants were told to rely on what they felt. The different videos were then presented to them in the experimental situation, and one video that never elicited any illusion was selected for the rest of the experiment.

In the fMRI experiment, there was a third condition in which the target stimulus was a white cross at the center of a black screen that was presented for 12 s (Fixation Cross condition).

### Experimental procedure

#### Training sessions

At the beginning of each training session participants watched their hand through the webcam. They simply moved their hand freely for a few minutes to adjust to the point of view. When they reported being sure that they were seeing their own hand on-line, a short video of their moving hand was recorded and the training started with this video and the chosen “other hand” video.

Participants were required to relax their body and focus their attention on the video.

Each session contained a total of four runs. Each run contained an identical number of Self Hand and Other Hand blocks (13 of each). Each block lasted 12 s and consisted of one video (self or other). Blocks were delivered in a pseudo-random order, with a 2 s mean inter-block interval (mean 2 s ± 1 s). The same condition never appeared more than twice in a row. Participants were instructed to press on the push button with their left hand to indicate the beginning of movement illusions in their right hand. During training there was constant visual monitoring of the participant's right hand to ensure it was not moving. In the early stages of training when the illusion started the hand started to move as well. Participants were told that they were moving their hand and they needed to keep it still. After only a few trials, this slight movement disappeared.

#### fMRI session

Participants laid on the bed of the MRI scanner. Their right hand was positioned in the exact position it was during the last training session using photographs taken during that session. They watched their hand on-screen using a MRI compatible camera that matched the point of view provided by the webcam during training. They freely moved their hand for a few minutes. When they reported being sure that they were seeing their own hand on-line, a short video of their moving hand was recorded. During the fMRI scanning sessions, participants were required to relax their body and focus their attention on the video or to fixate the white cross. To avoid any confounding activation within the sensorimotor regions there was no response to indicate the beginning of the illusion. We did not monitor real movement of the hand during fMRI, but in the later stages of training, the participant's hand never moved.

Each session contained a total of five runs. Each of the first four runs contained an identical number of Self Hand, Other Hand and Fixation Cross blocks (13 of each). Each block lasted 12 s and consisted of passive viewing of one video (self or other) or of 12 s presentation of the fixation cross. Blocks were delivered in a pseudo-random order, with a 2 s mean inter-block interval (mean 2 s ± 1 s). The same condition never appeared more than twice in a row. In the fifth run (localizer run), participants watched a red fixation cross. When the cross turned green participants had to perform very slow extension-flexion movements with their right hand. Each block of movement (green cross) lasted 12 s. There were 16 blocks of movement. The mean interblock interval (red cross) was 15 seconds, with a 4 s jitter. This real movement run was always performed last.

After the training and after the fMRI experiment participants filled a questionnaire including visual analog scales to assess the strength and consistency of body ownership, as well as the vividness of the kinesthetic sensation. The visual analog scale for rating kinesthetic sensation had 5 steps from “I didn't feel any movement” to “It felt exactly like a real movement” (0 to 100 mm).

#### MRI scanner and scanning sequences

Scanning was performed using a 3T whole-body imager MEDSPEC 30/80 AVANCE (Bruker, Ettlingen, Germany) equipped with a circular polarized head coil. High-resolution structural T1-weighted images were acquired from all participants for anatomical localization (MPRAGE sequence, 1*1*1 mm voxels). The anatomical slices covered the whole brain and were acquired in the sagittal plane. The functional images were acquired using a T2*-weighted echo-planar sequence with 42 axial slices (repetition time = 2.8 s, interleaved acquisition, slice thickness: 3 mm, field of view = 19.2 x 19.2 cm, 64 x 64 matrix of 3 x 3 mm voxels). The slices were parallel to the AC-PC plane and covered the whole brain. Participants were studied in four functional runs of 202 scans and one functional run of 146 scans, with a total duration of 45 minutes. For each run, the scanner was in the acquisition mode for 14 s before the experiment to achieve the steady-state transverse magnetization.

#### Image analysis

Data from one participant had to be discarded due to technical problems with the MRI scanner. Analyses reported here were performed on data from the 14 remaining participants.

Statistical parametric mapping software was used for image processing and analysis (SPM 08, Wellcome Department of Cognitive Neurology, London, UK). The functional images were interpolated in time to correct phase advance during volume acquisition and were spatially realigned to the first image of each run using trilinear interpolation. To allow multi-subject analysis, the anatomical references and the realigned functional images of all participants were transformed into a common standard space by using the MNI template. The functional data were then spatially smoothed (3D Gaussian kernel: 6 x 6 x 6 mm) and temporally filtered using a 128 s period high-pass filter. For each participant, two general linear models were applied to the time course of the functional signal at each voxel: One for the first four runs, and a separate one for the fifth run (localizer run). Each condition was modeled by a 12 s box-car function synchronized with the individual trials of this condition and convolved with a canonical hemodynamic response function.

Statistic parametric maps were calculated for individual T-contrasts for Self Hand vs Fixation Cross, Other Hand vs Fixation Cross and Self Hand vs Other Hand. The 14 images for each individual contrast (corresponding to the 14 participants) were then taken to 3 second level one-sample T-tests, one for each contrast. A T contrast was also calculated for each subject for Real Movement vs baseline (green cross vs red cross in the last run), and a second level T test was calculated.

The results were reviewed with the threshold of significance for active voxel set at p < 0.001 uncorrected and only clusters with p < 0.05 family wise error corrected for multiple comparisons at cluster level are reported here. Anatomical correspondence of significant clusters was double-checked using both the Anatomy toolbox [34–36] and the Anatomical Automatic Labeling toolbox [37].

For each subject individual Regions of Interest (ROIs) were defined using their own Real movement vs Baseline contrast. ROIs were: Primary Motor cortex (M1), Ventral Premotor cortex (PMv), Dorsal Premotor cortex (PMd) and Supplementary Motor Area (SMA). The individual Real movement vs Baseline contrasts were viewed with the threshold of significance for active voxel set at p < 0.001 uncorrected. The clusters over the regions of interest were identified by visual inspection and double checked using the Anatomy toolbox [34–36]. The Ventral Premotor cortex could not be reliably identified in some participants using this active movement localizer, and was therefore not included in the ROI analysis. The coordinates for the peak activation for each cluster were noted and used to build ROIs using the Marsbar software [38] as 10 mm diameter spheres. The Marsbar sofware was also used to calculate the percent signal change in each individual ROI for the three conditions: Self Hand, Other Hand, and Real Movement. The measures were entered in a 3 x 2 factorial analysis, with factors ROI (3 levels: M1, PMd, SMA) and Identity (2 levels: Self, and Other).

## Results

### Behavioral data

During the training sessions performed before the fMRI session, participants’ perception was assessed while they were watching videos of their own hand or of someone else’s hand presented in a random order. They were asked to press a button at the onset of any illusory perception. For all 15 participants included in the experiment, watching the video of their own moving hand resulted in an illusory kinesthetic sensation that their static hand was actually moving throughout the video presentation whereas such illusion was never elicited during the video of other hands. At the end of the training sessions (from 2 to 8 sessions according to the participant), the mean latency of the elicited illusions was 1.74 (± 0.38 SD) seconds.

**Fig 2:**
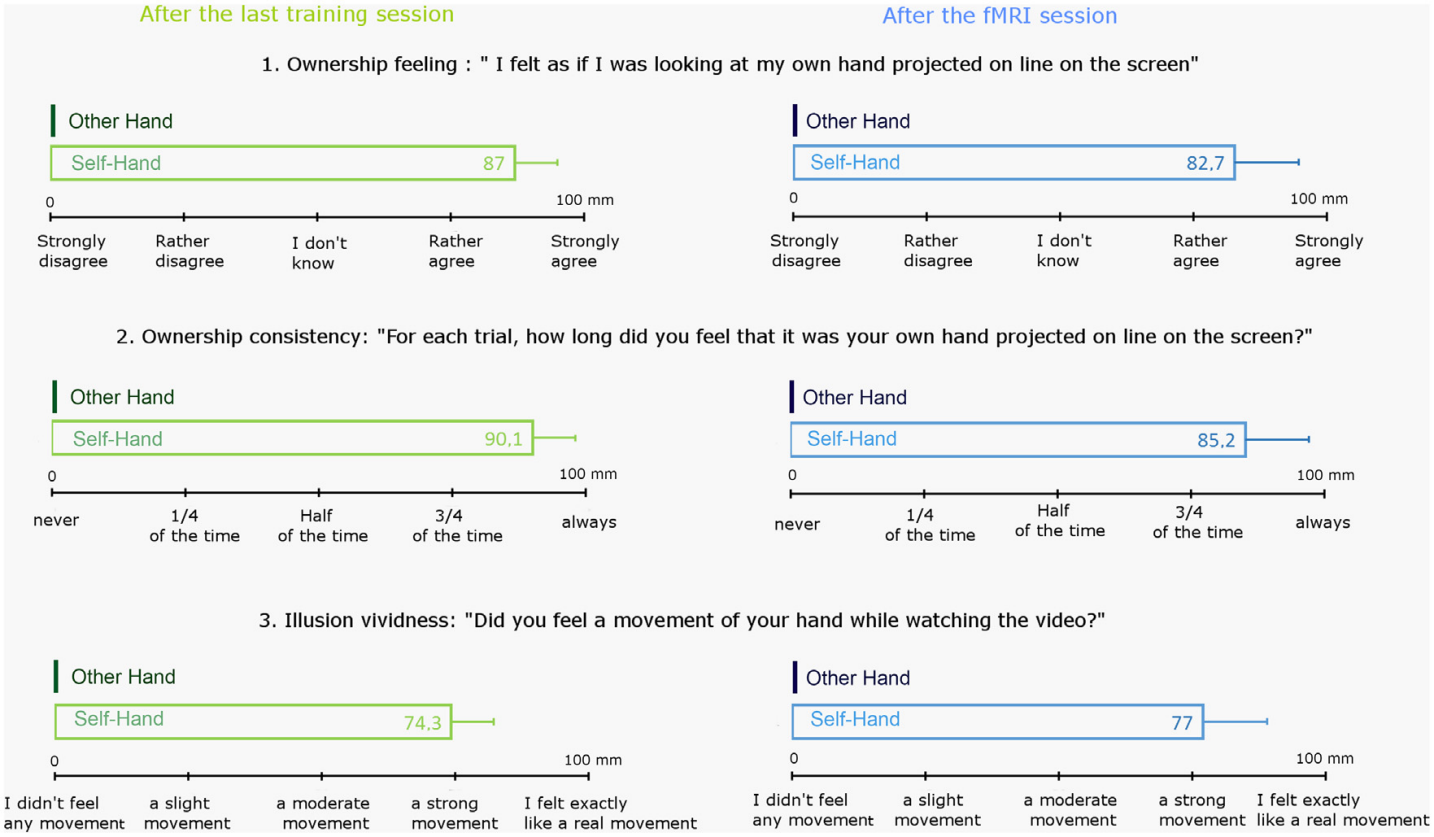
Responses to the questionnaire that participants filled after the last training session (green) and after the fMRI session (blue) to assess ownership feeling over the video projected hand (question 1), the consistency of ownership feeling (question 2), and the vividness of the kinesthetic sensation (question 3). Histograms are mean responses (+ standard deviation) reported by all participants on 100 mm analogue scales. It is important to note that the Other Hand video was specifically selected so it did not induce any feeling of ownership nor any sensation of movement (cf methods section).

Results for the questionnaire are summarized in Fig 2.

Subjective feelings were assessed using 100 mm visual analog scales with 5 graduations to help them. To assess ownership feeling participants had to indicate if they agreed or disagreed with the sentence “I felt as if I was looking at my own hand projected on line on the screen”. Graduations were “strongly disagree” (0 mm), “rather disagree” (25 mm), “I don't know” (50 mm), “rather agree” (75 mm) and “strongly agree” (100 mm). Response for Self Hand was 87 mm ± 9.9 mm (mean ± standard deviation, m ± sd) after the last training session and 82.7 mm ± 18.7 mm (m ± sd) after the fMRI session. To assess ownership consistency participants had to answer the question “For each trial, how long did you feel that it was your own hand projected on line on the screen?”. Graduations were “never” (0 mm), “1/4 of the time” (25 mm), “1/2 of the time” (50 mm), “3/4 of the time” (75 mm) and “always” (100 mm). Response for Self Hand was 90.1 mm ± 10.3 mm (m ± sd) after the last training session and 85.2 mm ± 12 mm (m ± sd) after the fMRI session. Finally, to assess the strength of the illusion participants had to answer the question “Did you feel a movement of your hand while watching the video?”. Graduations were “I didn't feel any movement” (0 mm), “I felt a slight movement” (25 mm), “I felt a moderate movement” (50 mm), “I felt a strong movement” (75 mm) and “It felt exactly like a real movement” (100 mm). Response for Self Hand was 74.3 mm ± 8 mm (m ± sd) after the last training session and 77 mm ± 12 mm (m ± sd) after the fMRI session.

In the Other Hand condition participants never reported feeling it was their own hand they saw, nor any illusory sensation of movement, but the hand used in this condition had been specifically selected so that the participants felt it could not be theirs and it did not induce illusory sensations of movement.

### Functional magnetic resonance imaging data

#### Random effect analyses

In the Self Hand vs Fixation Cross contrast, we found activation in a wide network of cortical and subcortical areas (Table 1). In the Other Hand vs Fixation Cross contrast, significant activation only appeared in the Occipital lobe, in bilateral Calcarine and lingual gyri, in right Frontal lobe, in the right Hippocampus and right Thalamus (Table 2).

**Table 1:** Self Hand > Fixation Cross. Results are given for each significant cluster including the global peak (first line) and local peaks. p values are corrected for familywise errors

Anatomic location	Peak MNI coordinates					
	x	y	z	Peak T value	voxels	p cluster
**Left Middle Occipital Gyrus**	**-30**	**-84**	**6**	**14.14**	**3292**	**<0.001**
Right Calcarine Gyrus	9	-90	0	10.76		
Right Middle Temporal Gyrus	48	-66	0	10.55		
Right Fusiform Gyrus	27	-81	-3	10.42		
Right Lingual Gyrus6	24	-84	-9	10.32		
**Left Precentral Gyrus**	**-33**	**-3**	**57**	**9.72**	**182**	**<0.001**
Left Superior Frontal Gyrus	-30	-9	69	4.64		
**Left Pars Opercularis**	**-57**	**9**	**15**	**9.10**	**106**	**<0.01**
Left Precentral Gyrus	-57	18	-3	5.63		
Left Rolandic Operculum	-48	3	9			
**Left Superior Parietal Lobule**	**-36**	**-54**	**60**	**8.43**	**257**	**<0.001**
Left Inferior Parietal Lobule	-33	-42	48	6.37		
Left Inferior Parietal Lobule	-60	-42	39	5.11		
**Right Pars Orbitalis**	**51**	**48**	**-6**	**6.78**	**49**	**<0.05**
Right Pars Triangularis	54	45	0	6.21		
Right Middle Orbital Gyrus	42	54	-9	4.22		
Right Middle Frontal Gyrus	51	48	9	4.21		
**Left Middle Frontal Gyrus**	**-36**	**45**	**0**	**6.25**	**60**	**<0.05**
Left Middle Orbital Gyrus	-42	51	-3	5.84		
**Right Insula**	**39**	**21**	**-3**	**6.24**	**219**	**<0.01**
Right Pars Opercularis	63	12	24	5.74		
**Right Caudate Nucleus**	**15**	**6**	**9**	**5.43**	**50**	**<0.05**
**Left Putamen**	**-21**	**6**	**6**	**4.95**	**68**	**<0.01**
**Left Cerebellum**	**-33**	**-63**	**-51**	**5.41**	**55**	**<0.05**
**Right Inf Parietal Lobule**	**36**	**-54**	**51**	**5.10**	**102**	**<0.01**
Right Sup Parietal Lobule	24	-66	60	4.90		
**Right Sup Temporal Gyrus**	**69**	**-36**	**21**	**4.96**	**55**	**<0.05**
Right Supramarginal Gyrus	60	-33	27	4.59		

**Table 2:** Other Hand > Fixation Cross. Results are given for each significant cluster including the global peak (first line) and local peaks. p values are corrected for familywise errors.

Anatomic location	Peak MNI coordinates					
	x	y	z	Peak T value	voxels	p cluster
**Right hippocampus**	**27**	**-30**	**-3**	**10.56**	**172**	**<0.001**
Right Thalamus	18	-27	0			
**Right Calcarine Gyrus**	**15**	**-93**	**6**	**11.00**	**3736**	**<0.001**
Right Lingual Gyrus	15	-87	-3	10.97		
Right Inf Occipital Gyrus	36	-84	-9	10.15		
Leftt Lingual Gyrus	-6	-84	-9	10.32		
Right Cuneus	18	-96	12	9.87		
**Left Thalamus**	**-18**	**-30**	**0**	**8.17**	**45**	**<0.05**
**Right Pars Opercularis**	**51**	**15**	**9**	**6.66**	**60**	**<0.01**
**Right Middle Orbital Gyrus**	**42**	**54**	**-9**	**6.34**	**55**	**<0.01**
Right Middle Frontal Gyrus	33	48	0	5.54		
Right Pars Orbitalis	54	42	-6	4.84		

**Fig 3:**
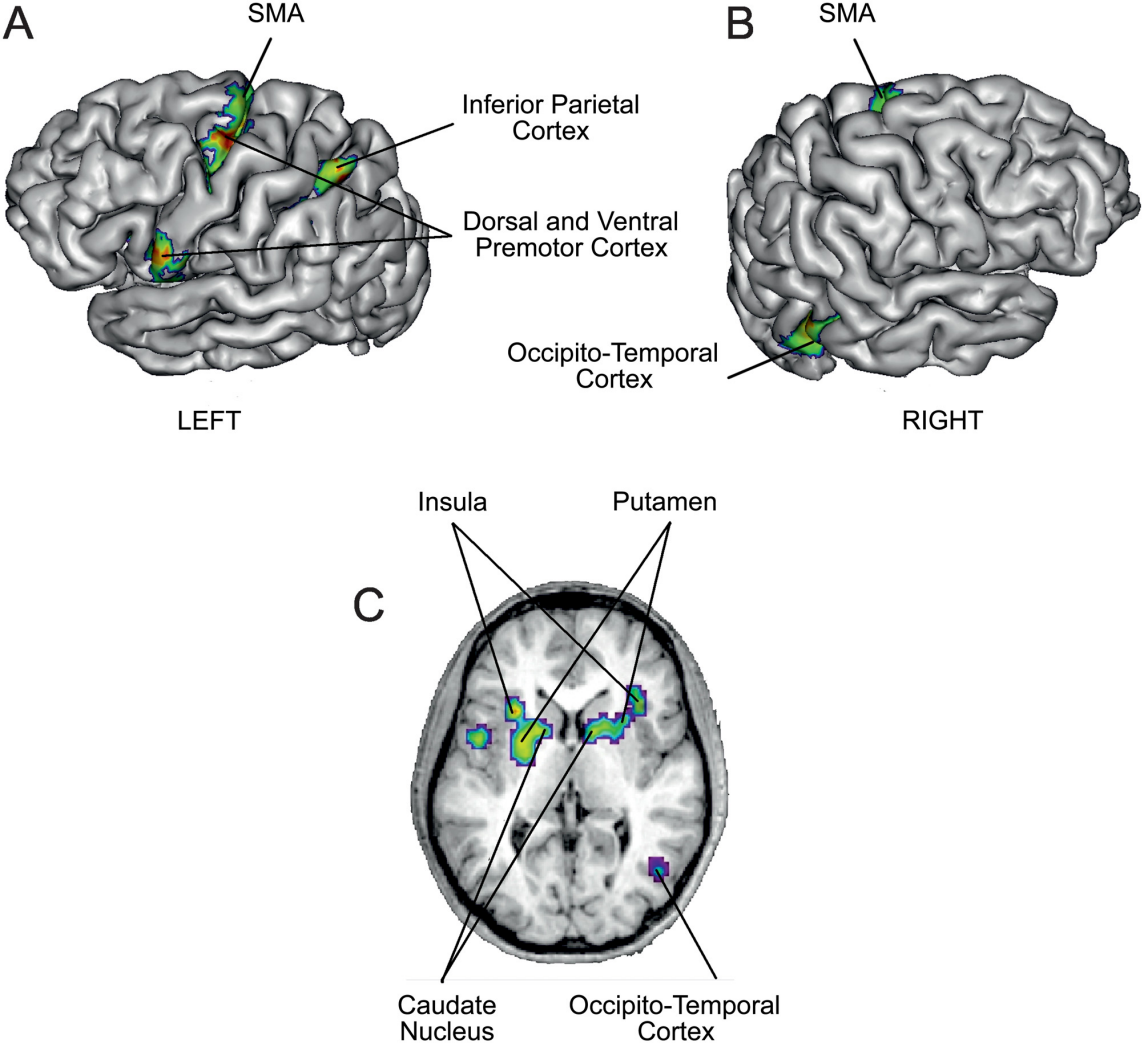
Self Hand > Other Hand. Voxel level threshold for both contrasts was set at 0.001 uncorrected and voxel level threshold was set at 0.05 FWE corrected. For visualization purpose, statistical maps are displayed on a single subject brain normalized onto the MNI template. Anatomical locations estimated using the Anatomy toolbox (Eickhoff et al. 2006; Eickhoff et al. 2007; Eickhoff et al. 2005) and the Anatomical Automatic Labeling (Tzourio-Mazoyer et al. 2002) are given in Table 3.

**Fig 4:**
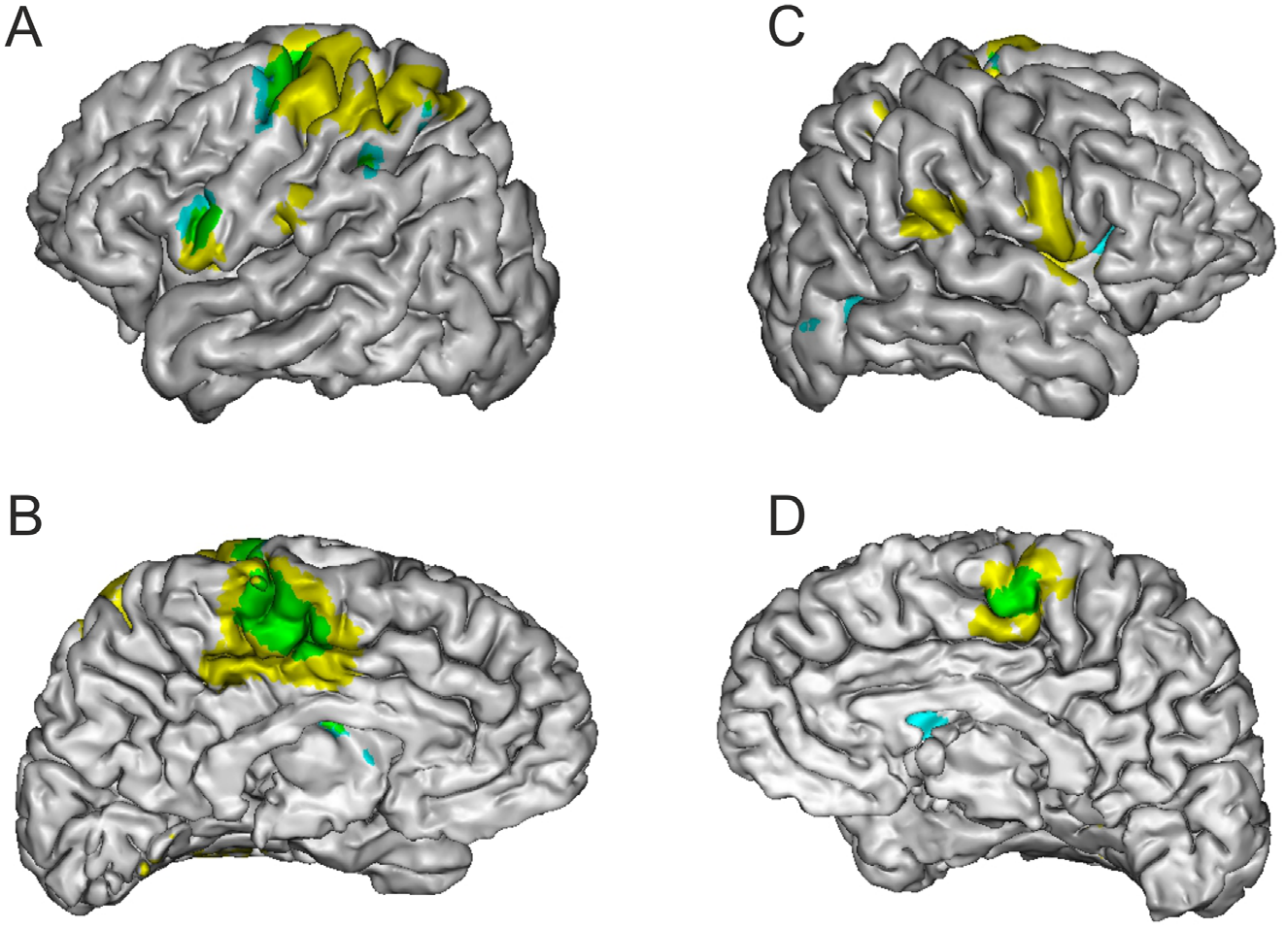
Statistical parametric maps for Self Hand > Other Hand and for Real movement > Baseline. Light blue = Self Hand > Other Hand; yellow = Real movement > Baseline; green = areas common to both contrasts. Voxel level threshold for both contrasts was set at 0.001 uncorrected and cluster level threshold was set at 0.05 FWE corrected. For visualization purpose, statistical maps are displayed on a single subject brain normalized onto the MNI template. Anatomical locations estimated using the Anatomy toolbox (Eickhoff et al. 2006; Eickhoff et al. 2007; Eickhoff et al. 2005) and the Anatomical Automatic Labeling (Tzourio-Mazoyer et al. 2002) are given in Tables 3 and 4.

In the Self Hand vs Other Hand contrast we found significant activation in the left Dorsal and Ventral Premotor cortices, in the left Intraparietal sulcus, at the right Occipito-Temporal junction as well as in bilateral Insula and Putamen (Table 3 and Fig 3). All areas activated in the Self Hand vs Other Hand contrast are also activated in the Real movement versus Baseline contrast except the right Occipito-Temporal junction (Table 4 and Fig 4). There was no activation over the primary motor and somatosensory cortices, even at lower threshold for cluster significance.

**Table 3:** Self Hand > Other Hand. Results are given for each significant cluster including the global peak (first line) and local peaks. p values are corrected for familywise errors.

Anatomic location	Peak MNI coordinates					
	x	y	z	Peak T value	voxels	p cluster
**Left Precentral Gyrus**	**-33**	**-3**	**60**	**7.24**	**377**	**<0.001**
Left Superior Frontal Gyrus	-24	-6	63	5.61		
**Left Superior Parietal Lobule**	**-36**	**-54**	**60**	**6.57**	**101**	**<0.01**
Left Inferior Parietal Lobule	-51	-42	45	4.94		
Left Inferior Parietal Lobule	-33	-45	51	4.74		
**Left Rolandic Operculum**	**-45**	**0**	**15**	**6.56**	**114**	**<0.01**
Left **Pars Opercularis**	-57	6	15	6.48		
**Left Middle Occipital Gyrus**	**-30**	**-84**	**6**	**14.14**	**3292**	**<0.001**
Right Calcarine Gyrus	9	-90	0	10.76		
Right Middle Temporal Gyrus	48	-66	0	10.55		
Right Fusiform Gyrus	27	-81	-3	10.42		
Right Lingual Gyrus6	24	-84	-9	10.32		
**Left Insula**	**-30**	**18**	**6**	**6.43**	**195**	**<0.001**
Left Putamen	-27	3	6	5.79		
**Right Fusiform Gyrus**	**30**	**-81**	**-9**	**5.97**	**116**	**<0.01**
Right Middle Temporal Gyrus	48	-69	3	5.91		
Right Inferior Temporal Gyrus	51	-63	-3	5.67		
Right Inferior Occipital Gyrus	39	-78	-3	4.73		
**Right Insula**	**39**	**21**	**3**	**5.39**	**94**	**<0.01**
Right Caudate Nucleus	12	6	6	5.22		
Right Putamen	30	15	3	4.91		

**Table 4:** Real Movement > Baseline. Results are given for each significant cluster including the global peak (first line) and local peaks.

Anatomic location	Peak MNI coordinates					
	x	y	z	Peak T value	voxels	p cluster
**Left Precentral Gyrus**	**-39**	**-27**	**57**	**14.14**	**1802**	**<0.0001**
Left Precentral Gyrus	-36	-12	54	11.89		
Left Postcentral Gyrus	-30	-39	57	11.79		
Right SMA	9	3	69	12.21		
Left SMA	0	-3	66	11.04		
**Left SMG**	**-54**	**-24**	**24**	**13.03**	**162**	**<0.0001**
Left Rolandic Operculum	-48	-30	21	8.02		
**Right Precentral Gyrus**	**63**	**9**	**21**	**10.22**	**318**	**<0.0001**
Right Rolandic Operculum	57	9	6	10.06		
Right Putamen	24	3	6	9.48		
Right Insula	39	-3	9	7.17		
Right Caudate	15	-3	18	3.90		
**Right SMG**	**60**	**-24**	**24**	**9.28**	**119**	**<0.0001**
Right Sup Temp Gyrus	60	-36	21	6.07		
**Left Insula**	**-33**	**0**	**9**	**8.34**	**443**	**<0.0001**
Left Putamen	-30	-6	3	7.88		
Left Rolandic Operculum	-51	0	3	7.84		
**Right Inf Parietal Lobule**	**39**	**-39**	**45**	**5.66**	**49**	**<0.01**
Right Sup Parietal Lobule	36	-45	57	4.75		
Right Postcentral Gyrus	45	-42	60	4.24		

#### Regions of Interest analyses

Before performing the ROI analysis we checked whether the PMd and SMA peaks in the Self Hand vs Other Hand contrast and in the Real Movement vs Baseline contrast were in comparable locations. For each subject the coordinates for the peak activation in left PMd and in SMA were measured for each contrast and later entered into two Hotelling's T^2^ tests for two multivariate dependent samples. Both tests were non significant although only marginally (T^2^ = 11.77; p = 0.061 for PMd and T^2^ = 9.79; p = 0.1 for SMA).

**Fig 5:**
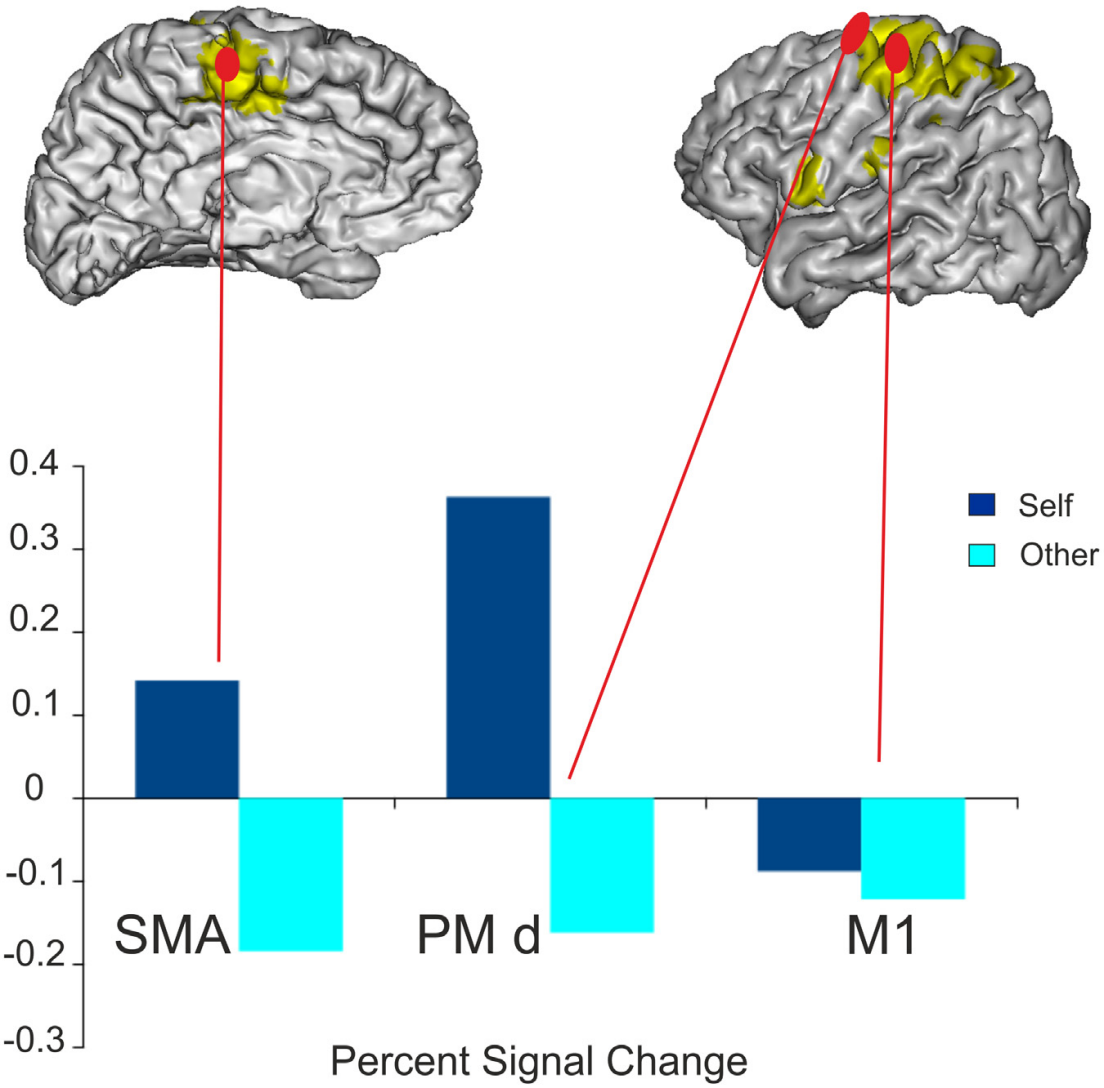
Percent Signal Change during the Self Hand and the Other Hand conditions for 3 motor Regions of Interest (ROIs). ROIs were defined for each subject from their individual Real movement > Baseline contrast. For visualization purpose the statistical parametric map for the group analysis of the Real movement > Baseline contrast is projected onto a single subject brain normalized onto the MNI template.

The 3 x 2 factorial analysis showed a significant interaction between factors ROI and Identity (F_2,26_ = 7.55, p = 0.0026). Post-Hoc tests showed that the percent signal change was significantly higher during the Self Hand condition than during the Other Hand condition in PMd and SMA but not in M1 (Fig 5). In the Other Hand condition the percent signal change was negative in all three ROIs, but in the Self Hand condition it was positive in PMd and SMA and slightly negative in M1 (means and standard deviations in Table 5).

**Table 5:** Percent signal change in M1, PMd and SMA during the Self Hand and the Other Hand conditions. Values are mean ± standard deviation.

	SMA	PMd	M1
*Self Hand*	0.142 ± 0.094	0.363 ± 0.185	-0.088 ± 0.080
*Other Hand*	-0.185 ± 0.093	-0.162 ± 0.104	-0.122 ± 0.060

## Discussion

We report cerebral activation patterns during kinesthetic perception of limb movements induced by visual stimulation. Both conditions in this study involved watching movies of slow wrist extension-flexion sequences. When the movie depicted the participant's self right hand (Self Hand condition) he/she felt the illusory sensation that his/her right hand was actually moving, whereas when the movie depicted someone else's hand (Other Hand condition) there was no kinesthetic perception. In the Self Hand vs Other Hand contrast activation was found in bilateral Supplementary Motor Area, Anterior Insula, Putamen, Caudate Nuclei, in left Inferior Frontal gyrus (Pars Opercularis), Dorsal Premotor cortex and Inferior Parietal cortex/Intraparietal sulcus, as well as at the right Occipito-Temporal junction and Fusiform gyrus. This network largely overlapped with the one evidenced while participants really performed slow wrist extension-flexion movements.

### Discussion in the context of kinesthetic illusions

The network of areas evidenced here is also compatible with cerebral activations evidenced during movement illusions induced by muscle proprioceptive [7,15–20] or tactile stimulation [7]. The main differences were that the primary motor and somatosensory cortices were not active during the present movement illusion, while the right Occipito-Temporal junction was not active during previously described movement illusions.

It was puzzling for us at first to note that M1 was not activated during this visually-induced kinesthetic illusion. A number of previous studies using PET, fMRI or MEG have demonstrated that primary motor (M1) is activated during kinesthetic illusions induced by proprioceptive afferent input and/or tactile stimulation [7,14–16,18–20]. A role for M1 activity in kinesthetic perception was also supported by a physiological study in which TMS applied to M1 and S1 was found to alter the vibration-induced kinesthetic illusion [39]. A previous MEG study using tendon vibration showed that M1 activation appeared very early in Illusion trials with a sharp increase around the time the illusion began, whereas there was no M1 activation at all during No Illusion trials [21]. Taken together those studies make a strong case for M1 playing an essential part in kinesthetic illusions. Moreover, in a previous study using TMS we found that the motor evoked potentials (MEPs) increased during a very similar visually-induced illusion, suggesting an increased excitability of the motor pathways [8,9]. We cannot rule out that the present experiment lacked power to detect M1 activation. But we could not find any cluster over the central sulcus in the group analysis, not even a non significant one. Also, the percent signal change analysis showed that the signal over M1 was not different during the Self Hand condition and the Other Hand condition. Moreover, if anything, the change in signal during these two conditions was slightly negative as compared to the mean level in this ROI during the whole experiment (i.e. including the fixation condition) that only consisted of passive viewing conditions.

The strong M1 involvement in previously studied movement illusions makes sense as those were induced by muscle proprioceptive or tactile afferent stimulation [7,14–18]. Indeed, a recent physiological study by Christensen et al. [40] reported that rTMS applied to M1 and to PMd both induced sensations of movement. Moreover, using nerve block that shuts proprioceptive afferent inflow, they showed that the sensation of movement induced by rTMS applied to M1 was significantly influenced by sensory feedback, indicating that M1 is associated with the processing of somatosensory afferent input. However, as reported by Christensen et al. [40], the sensation of movement induced by PMd stimulation was not significantly influenced by sensory afferent input; therefore the authors proposed that rTMS applied over PMd produces a corollary discharge that is perceived as movement. Furthermore, Desmurget et al. [41] reported that direct cortical electrical stimulation of the Inferior Parietal cortex (BA40 and 39) induced movement perception. In their study, participants believed they had actually performed the movements. Our study revealed significant activation in both the PMd and Inferior Parietal cortex during kinesthetic perception. In a previous study, Christensen et al. [40] suggested that movement perception could arise not only from sensory processing but also from top-down processes within a network comprised of areas involved in motor planning and in sensory-motor integration. It is one possible explanation for the present report of a movement illusion subtended by a largely parieto-premotor network, with no M1 activation. The seemingly contradictory results from our previous experiment were TMS evoked MEPs were modulated during a similar visually-induced illusion [8] might have several explanations. First as the TMS was not MRI-guided, it is possible that the localization of M1 was slightly imprecise and that the TMS site also covered part of PMd. It is also possible that the excitability of the motor pathways was modulated below M1 by efferents from the premotor areas that are activated during the illusion.

The proposition that movement perception could arise from top-down processes within a network comprised of areas involved in motor planning [40] can find some support in motor imagery studies. Indeed motor imagery is an internal simulation of action, without actual motor execution. It thus involves motor planning and prediction of sensory consequences of the planned action. In a recent meta-analysis Hétu et al. [42] found a wide array of Fronto-Parietal regions, including PMd, SMA, Inferior Frontal gyrus, Rolandic Operculum, Inferior Parietal Lobulus, Supramarginalis gyrus, Angular gyrus, as well as Temporal pole, Anterior Insula, Putamen and Cerebellum [42]. With direct comparison between motor imagery and motor execution, Hanakawa et al. [43,44] defined three types of brain regions. Type I areas that included M1 and S1 were mainly activated in motor execution. Type III areas (rostral PMd, rostral SMA and Frontal Eye Fields) were mainly activated in motor imagery. Type II areas were equally activated during motor execution, motor imagery and motor planning. They included Dorsal and Ventral Premotor cortices, SMA proper, Posterior Parietal cortex (Superior and Inferior lobules), and Inferior Frontal gyrus (Operculum). The cortical activations we describe here are in type II regions that is regions more involved with pre-executive processes than with motor execution [44].

Another puzzling finding from this study might be explained by motor imagery studies. Indeed, here we did not find M1 activation even when the Self Hand and Other Hand conditions were each contrasted with the fixation condition whereas M1 activation has been reported during action observation tasks [45–51]. So one could expect M1 activation in both contrasts as “fixation” is a very low level control condition. However, the participants that participated in this study underwent a thorough training in which they were repeatedly instructed to keep their arm and hand very relaxed while they experienced the illusion. Each training block was preceded by the reminder that they should not move. Several motor imagery studies have shown that in good (or expert) imagers M1 activation was lower than in bad (or non-expert) imagers [52–54]. This finding is reinforced by effective connectivity studies that show that during motor imagery SMA has an inhibitory effect on M1 [55–57]. In motor imagery tasks, participants are required to mentally simulate an action, without performing it. So one possibility is that part of becoming an expert imager is learning to suppress executive processes. Likewise it is possible that while they were trained to feel that illusion better while keeping their hand very still, our participants learned to suppress those executive processes, which would explain why we do not get M1 activation even with a very low level control condition.

### Discussion in the context of body ownership

In this experiment the movement illusion was induced using videos of the self hand while the control condition presented videos of someone else's hand. Moreover participants reported a sense of ownership over the video-projected hand only when it was their own hand. There could therefore be some confounding effect between movement illusion and self/other discrimination and/or body ownership. The right Occipito-Temporal region is active when participants watch both videos (Self Hand and Other Hand), but it is more active for the Self Hand video than for the Other Hand video. This activation in right Occipito-Temporal cortex is compatible with the known location of the Extrastriate Body Area (EBA, [58]) that responds to the vision of body parts. It has been proposed that EBA also participates in the discrimination between self and other body parts [59–61]. It is therefore likely that the activation we report here is indeed located in the EBA.

It has also been suggested that left premotor cortex could also discriminate self from other's right hand, and that bilateral Insula and SMA were also involved in general self/other discrimination [61]. More generally, it has been argued that body ownership relies on multisensory integration in peripersonal space, subtended by premotor-parietal networks [62–66], with participation from Putamen [65,67] and posterior Insula [66–69]. So the entire network described in the present study has been linked to Self/Other discrimination and/or body ownership, with the exception of the insula. Indeed the anterior activation we report here is more in agreement with activation during movement illusions [7]. In recent years the question of body ownership has been addressed using body illusions such as the rubber hand illusion [64,70–73]. In the classical rubber hand illusion, synchronous cutaneous stimulation is applied over the participant's unseen hand and a seen rubber hand, which leads the participant to misperceive his/her unseen hand as being closer to the rubber hand than it really is. This error in felt position is believed to result from embodiment of the rubber hand on the basis of multisensory integration of visual, cutaneous and proprioceptive information [64,70–73]. It has been proposed that an internal body model is also needed and that the seen object can only be embodied if it fits with this internal body model [71–74]. Rubber hand illusions can also be induced using movement stimulation. In these situations active or passive movement of the unseen real hand while the participant watches a model hand move synchronously induces a shift in the perceived location of the real hand toward the location of the model hand [75–78]. Similar illusions can also be induced when the participants watch video-projected movements of their own hand, while their unseen hand moves, actively or passively, in synchrony with the video-projected hand [79–81]. The key to these different forms of “moving” rubber hand illusions is the synchrony between the real movement of the hand and the movement of the model or video-projected hand. Recently Kalckert and Ehrsson [77] compared visuotactile and movement induced illusions. They concluded that they were essentially the same illusion, and that different combinations of information could lead to the same changes in ownership perception. They suggested that the rubber hand illusion does not depend on specific sensory signals, but that it rather depends on the spatiotemporal relationship of the signals available. In the present experiment, we do not have another sensory input synchronous to the movement of the video-projected hand, so multisensory integration relies on the internal body model, the visual and the proprioceptive inputs. When the hand on the screen is the participant's own, the fit with the internal body model is very high, the seen and the felt positions are the same which would lead to the illusory feeling that the hand is moving. In agreement with this suggestion that the kinesthetic illusion in this study depends on the same mechanisms of sensorimotor integration than the rubber hand illusions is the fact that 70.3% of the participants we tested experienced this illusion. That is similar to the rate of responders for all the forms of rubber hand illusions [64,76,77].

One could argue that as the participants felt their hand moving, they could experience a sense of agency. We did not test the participants for a sense of agency because in their spontaneous reports in the first stage of the training they never claimed they caused the movement (“My hand wants to move”, “I don't want my hand to move, yet it moves”, “My hand moves, are you moving it?”). Still we cannot rule this possibility out even though there was neither motor command nor motor intention in our situation. For example, Longo and Haggard [81] studied ownership and agency while participants viewed videos of their own hand on a screen in several conditions. They showed that the sense of ownership was present in all conditions when the stimulus on screen was synchronous with the stimulus on the real hand, while the sense of agency only arose for synchronous active movement. Kalckert and Ehrsson [76] reported similar findings for rubber hand illusions evoked with moving model hands. But we do agree with Kalckert and Ehrsson hypothesis that ownership may facilitate agency over bodily actions [77], so since our participants felt ownership over the video-projected hand it is possible that it lead to some degree of latent agency: If it is their hand, then they could move it should the need arise.

## Conclusion

Although we argue that the mechanisms that induce the present illusion could be partly the same that lead to the rubber hand illusion, the illusions themselves are different. In the present study participants report the feeling that their hand is moving. The illusion that the hand is moving starts about 2 seconds after the onset of the video and lasts till the end of the video. So this illusion more resembles kinesthetic illusions previously described during tendon vibration, tactile and/or visual stimulation [1,2,6–11,16].

We propose that multimodal integration of sensorimotor and spatial information with internal body models lead to ownership of the video projected hand. This in turn could lead to activation of a network that is usually involved in processing copies of efference, thus leading to the illusory perception that the real hand is indeed moving. This approach is of great interest to assess the involvement of visual information in self body movement perception. Although the benefit of the mirror paradigm to promote recovery is still debated and some authors suggested that bimanual coupling in the mirror paradigm might be the key factor in rehabilitation [82,83], the present paradigm might be proposed to patients who have both hands immobilized since it does not require that one hand actually moves to induce the illusion. As shown by our previous work [8], the hand on the video does not need to be the patient's own hand, as long as it does not look too different from the patient's.
